# HMGCR and Rosuvastatin Regulates GLP-1 Secretion and Expression—A Translational Study

**DOI:** 10.1210/clinem/dgaf608

**Published:** 2025-11-05

**Authors:** Michael G Miskelly, Andreas Lindqvist, Amra Jujić, Alexander Hamilton, Elaine Cowan, Sweta Raikundalia, Anna-Maria Dutius Andersson, Bent J Nergård, Rita Del Giudice, Dmytro Kryvokhyzha, Peter M Nilsson, Jens Juul Holst, Signe Sørensen Torekov, Jens O Lagerstedt, Maria F Gomez, Lena Eliasson, Hindrik Mulder, Jan Hedenbro, Martin Magnusson, Nils Wierup

**Affiliations:** Neuroendocrine Cell Biology, Department of Experimental Medical Science, Lund University, Malmö 21428, Sweden; Neuroendocrine Cell Biology, Department of Experimental Medical Science, Lund University, Malmö 21428, Sweden; Department of Clinical Sciences, Lund University, Malmö 21428, Sweden; Unit of Molecular Metabolism, Lund University Diabetes Centre, Malmö 21428, Sweden; Islet Cell Exocytosis, Lund University Diabetes Centre, Lund University, Malmö 21428, Sweden; Islet Cell Exocytosis, Lund University Diabetes Centre, Lund University, Malmö 21428, Sweden; Neuroendocrine Cell Biology, Department of Experimental Medical Science, Lund University, Malmö 21428, Sweden; Diabetic Complications Unit, Department of Clinical Sciences, Lund University Diabetes Centre, Malmö 21428, Sweden; Aleris Obesitas, Lund 22270, Sweden; Department of Experimental Medical Science, Lund University, Lund 22100, Sweden; Department of Biomedical Science and Biofilms–Research Center for Biointerfaces, Malmö University, Malmö 21432, Sweden; Department of Clinical Sciences, Lund University, Malmö 21428, Sweden; Department of Clinical Sciences, Lund University, Malmö 21428, Sweden; Department of Biomedical Sciences, Faculty of Health and Medical Sciences, The Panum Institute, University of Copenhagen, Copenhagen 2200, Denmark; Novo Nordisk Foundation Center for Basic Metabolic Research, Faculty of Health and Medical Sciences, University of Copenhagen, Copenhagen 2200, Denmark; Department of Biomedical Sciences, Faculty of Health and Medical Sciences, The Panum Institute, University of Copenhagen, Copenhagen 2200, Denmark; Islet Cell Exocytosis, Lund University Diabetes Centre, Lund University, Malmö 21428, Sweden; Department of Experimental Medical Science, Lund University, Lund 22100, Sweden; Diabetic Complications Unit, Department of Clinical Sciences, Lund University Diabetes Centre, Malmö 21428, Sweden; Islet Cell Exocytosis, Lund University Diabetes Centre, Lund University, Malmö 21428, Sweden; Unit of Molecular Metabolism, Lund University Diabetes Centre, Malmö 21428, Sweden; Neuroendocrine Cell Biology, Department of Experimental Medical Science, Lund University, Malmö 21428, Sweden; Department of Surgery, Lund University, Lund 22185, Sweden; Department of Clinical Sciences, Lund University, Malmö 21428, Sweden; Department of Cardiology, Skåne University Hospital, Malmö 21428, Sweden; Neuroendocrine Cell Biology, Department of Experimental Medical Science, Lund University, Malmö 21428, Sweden; Department of Experimental Medical Science, Lund University, Lund 22100, Sweden; Clinical Research Centre, Skåne University Hospital, Malmö 21428, Sweden

**Keywords:** GLP-1, glucose homeostasis, HMGCR, incretins, rosuvastatin, type 2 diabetes

## Abstract

**Context:**

Statin use is associated with increased risk of type 2 diabetes (T2D) and mild hyperglycemia. The underlying mechanisms are not well studied, and the effect of statin treatment on glucagon-like peptide 1 (GLP-1) secretion or production is unknown.

**Objective:**

This work aimed to assess the effects of rosuvastatin on GLP-1 secretion and production.

**Methods:**

We performed association studies in the Malmö Diet and Cancer study cardiovascular cohort (MDCS-CC) reexamination cohort, in vitro investigations using GLUTag cells and acute and chronic studies in female, normoglycemic C57Bl/6j mice.

**Results:**

Studies in the MDCS-CC reexamination cohort (n = 3734) revealed that in individuals without T2D, statin usage was associated with higher fasting glucose-dependent insulinotropic peptide (GIP), insulin, glucose, glucagon, and homeostatic model assessment of insulin resistance, but not GLP-1. However, in patients with T2D, statin usage was associated with higher fasting GLP-1 levels. Rosuvastatin treatment or 3-hydroxy-3-methyl-glutaryl-coenzyme A reductase (*Hmgcr*) knockdown (KD) reduced GLP-1 secretion and increased *Gcg* messenger RNA in GLUTag cells. Rosuvastatin acutely reduced postprandial GLP-1 secretion, whereas chronic rosuvastatin treatment in mice caused hyperglycemia and increased postprandial GLP-1 levels. The acute effect of *Hmgcr* KD on GLP-1 secretion could be mimicked by targeting intracellular cholesterol using a PCSK9 inhibitor. Finally, transcriptomic alterations induced by rosuvastatin were limited to genes involved in cholesterol biosynthesis.

**Conclusion:**

We have established HMGCR as a regulator of GLP-1 secretion and provide a plausible explanation for the clinically observed mild hyperglycemia associated with statin use. Given the negative acute effect on GLP-1 secretion, monitoring of blood glucose levels is recommended after prescribing rosuvastatin.

The beneficial effects of statin treatment on mortality are unquestionable (1, 2). However, several studies have associated increased risk of type 2 diabetes (T2D) with statin usage (3) and the hyperglycemic actions of statin treatment are well known (4). Although both clinical and population-based studies suggest that the cardiovascular benefits of statin therapy outweigh the risk of developing T2D (5, 6), other studies pinpoint the need to closely monitor statin use in high-risk groups being assessed for perturbed glucose homeostasis (7, 8).

Given the mounting evidence that statin treatment increases the risk of transition to T2D in individuals with impaired glucose metabolism (3, 7), the lack of studies on the mechanisms involved is surprising. Nevertheless, Abbasi and colleagues (9) recently reported data from an open-label clinical trial of 40-mg daily atorvastatin in adults without known atherosclerotic cardiovascular disease or T2D at baseline. The researchers’ findings showed that treatment for 10 weeks increases insulin resistance and secretion; this urges for further investigation into the mechanisms driving these effects. Furthermore, studies on how statin treatment affects other well-known glucoregulatory hormones, such as glucagon-like peptide 1 (GLP-1), glucose-dependent insulinotropic peptide (GIP), and glucagon, are, to the best of our knowledge, lacking.

There are reports showing that 3-hydroxy-3-methyl-glutaryl-coenzyme A reductase (*HMGCR*) (encoding the target for statins) genetic variants are associated with impaired fasting glucose and increased risk of diabetes (10, 11). Based on this, combined with the reported effects of statins on glycemia, we hypothesized that statins affect GLP-1 secretion and production. The effects of statin treatment on glucose, insulin, and GLP-1 were examined in experimental models and associations between statin use and circulating levels of glucoregulatory hormones were explored in a large Swedish population-based cohort.

## Materials and Methods

### Participants

The Malmö Diet and Cancer Study (MDCS) included 30 447 participants in Malmö, Sweden, with baseline assessments conducted from 1991 to 1996. A subcohort (n = 6103) formed the MDCS Cardiovascular Cohort (MDCS-CC) to study cardiovascular risk factors. Within this cohort, 3734 individuals underwent reexaminations, including oral glucose tolerance test (OGTT), between 2007 and 2012, forming the study population for this analysis (12).

### Clinical Assessment

Participants underwent fasting blood sampling and anthropometric measurements. T2D was defined by physician diagnosis, antidiabetic medication use, or OGTT criteria (fasting plasma glucose [FPG] ≥7.0 mmol/L or ≥12.2 mmol/L post OGTT). Statin use was assessed via the Swedish Prescribed Drug Registry (13).

### Oral Glucose Tolerance Test in Participants

A 75-g OGTT (14) was conducted after an overnight fast, with blood samples collected at 0, 30, and 120 minutes for glucose, insulin, glucagon, GLP-1, and GIP. Individuals with diabetes did not undergo OGTT.

### Laboratory Assays

Samples for GIP were collected in serum tubes with a clot activator and stored at −20 °C, while GLP-1 samples included a dipeptidyl peptidase 4 inhibitor and were stored at −80 °C. GLP-1 was measured by radioimmunoassay (RIA; detection limit 1 pmol/L, coefficient of variation <6% intra-assay, < 15% interassay; RRID: AB_2892837) at Copenhagen University (15). GIP was analyzed using Human GIP Total ELISA (enzyme-linked immunosorbent assay; EZHGIP-54 K, MilliporeSigma; RRID: AB_2801401). FPG was measured with the HemoCue Glucose System, serum insulin with a Dako ELISA (RRID: AB_2935709), and glucagon with an RIA GL-32 K (MilliporeSigma; RRID: AB_2757819). Insulin resistance was calculated via the homeostatic model assessment of insulin resistance (HOMA-IR). High-density lipoprotein cholesterol and triglycerides were analyzed according to standard procedures at the University Hospital Malmö, following national quality standards.

### Immunohistochemistry

Immunohistochemistry was performed as described previously (16) using primary antibodies for rabbit anti-HMGCR (dilution 1:200, SAB4200528, Sigma Aldrich, RRID: AB_3712669) and guinea pig anti-glucagon (1:2500, M8707, Euro-Diagnostica, RRID: AB_3713051). The secondary antibodies (dilution 1:400) used were CyTM2 Affinipure donkey anti-rabbit IgG (711225152; RRID: AB_2340612) and CyTM5 Affinipure donkey anti-guinea pig IgG (706175148; Jackson ImmunoResearch Europe; RRID: AB_2340462). Primary and secondary antibodies were diluted in 0.25% bovine serum albumin and 0.25% Triton X-100 in phosphate-buffered saline. The HMGCR antibody was validated by staining in mouse liver as a positive control as well as a negative control (0.25% bovine serum albumin and 0.25% Triton X-100 without the antibody).

### Cell Culture

GLUTag cells (RRID: CVCL_J406) were kindly provided by Prof D. J. Drucker, Mount Sinai Hospital, Toronto, Canada, and used for gene expression, secretory, intracellular signaling, glucose uptake, ApotoxGlo Triplex, and cholesterol efflux assays. Cells were cultured as described previously (17). Cells were tested for mycoplasma contamination using the Lonza MycoAlert Mycoplasma Detection Kit (LT07-418, Lonza).

### Small Interfering RNA-mediated Gene Silencing

GLUTag cells were seeded in 24-well plates with 250 000 cells per well. *Hmgcr* knockdown (KD) was performed using Lipofectamine RNAiMAX (Life Technologies) and a small interfering RNA (siRNA) targeting *Hmgcr* (s67592; Silencer Select Pre-designed siRNA; Ambion, Life Technologies) in the mouse genome that was transfected at 50 pmol/L as per the manufacturer's protocol. Scrambled siRNA (4390844) was used as a negative control (Silencer Select Negative Control No. 1 siRNA: Ambion, Life Technologies). RNA was extracted 48 hours after KD as per the manufacturer's instructions (NucleoSpin RNA II, Macherey Nagel).

### Incubation of GLUTag Cells with Rosuvastatin and Bulk RNA Sequencing

GLUTag cells seeded on 24-well plates as described earlier were incubated at 37 °C 5% CO_2_ for 24 hours before being treated with rosuvastatin (20 nmol/L to 2 µmol/L) in normal growth media for 24 hours. Untreated cells were provided with fresh normal growth media. RNA was extracted from the cells as per manufacturers’ instructions (NucleoSpin RNA II, Macherey Nagel). Following extraction, RNA was either converted for quantitative real-time polymerase chain reaction (qPCR) or bulk RNA sequencing (RNA-seq). RNA libraries were generated using the Illumina TruSeq Stranded Total RNA Human (Illumina) protocol following the manufacturers’ recommendations.

### Glucose Uptake, Apoptosis, Cell Viability, and Cytotoxicity

Cells were seeded on 96-well plates at a density of 20 000 cells per well. siRNA-mediated KD was performed as described earlier and glucose uptake was measured using the Glucose Uptake-Glo assay (Promega) as per the manufacturer's instructions. Apoptosis, cell viability, and cytotoxicity were measured using an Apotox-Glo Triplex assay (Promega) as per the manufacturer's instructions.

### Gene Expression Analysis

RNA was reverse-transcribed using RevertAid First Strand complementary DNA synthesis kit (Thermo Scientific). qPCR for *Dpp4* (Mm00494538_m1), *Gip* (Mm00433601_m1) *Hmgcr* (Mm01282499_m1), *Proglucagon* (*Gcg*; Mm01269055_m1), *Pcsk1* (Mm00479023_m1), *Pyy* (Mm00520715_m1), and 2 housekeeping genes (*Hprt* [Mm03024075_m1] and *Tbp* [Mm01277042_m1]) was performed using TaqMan Expression PCR Master Mix (Life Technologies) using the ABI Prism 7900 HT system (Applied Biosystems). Data analysis was carried out using the 2^−ΔΔCt^ method. RNA-seq libraries were generated using the Illumina TruSeq Stranded Total RNA Prep with Ribo-Zero Plus protocol (Illumina) and sequenced with the Illumina NextSeq 500/550 High Output Kit v2.5 on an Illumina NextSeq 500 instrument (75 bp, paired end). Sequence quality was evaluated using FastQC 0.11.9 (18) and summarized with MultiQC 1.10.1 (19). The differential gene expression analysis was conducted using DESeq2 v1.30.1 (20) on the count matrix generated by Salmon v1.5.0 (21). Only genes with a minimum median expression of 3 reads in at least 1 comparison group were included in the analysis. *P* less than .05 was used as a statistical significance threshold. GO-term analysis on nominally significant genes was performed using PANTHER version 19.0 overrepresentation test.

### Secretion Assays

Secretory assays in response to glucose and KCl were performed as described previously (17, 22). Briefly, GLUTag cells were seeded, and siRNA-mediated KD was performed as described earlier with secretory assays being performed 72 hours post seeding. GLUTag cells for rosuvastatin treatment were seeded and incubated at 37 °C 5% CO_2_ for 48 hours. Cells were then treated for 24 hours before the assay. The collected supernatant was frozen at −20 °C in preparation for GLP-1 measurement. Concentrations of GLP-1 were determined using the Multi Species GLP-1 Total ELISA (EZGLP1T-36 K, MilliporeSigma; RRID: AB_2813786) as per the manufacturer’s instructions.

### Intracellular Calcium Imaging

GLUTag cells were plated on poly-D-lysine-coated (1μg/mL) Lab-Tek 8-well chambered coverglass dishes (155411; Thermo Scientific) at a density of 60 000 cells/well, using only the 4 middle wells. Cells were incubated for 24 hours before treatment with 2-µM rosuvastatin. Measurements of [Ca^2+^]_c_ were performed as described previously (22).

### Cholesterol Efflux

GLUTag cells were plated at a density of 150 000 cells per well in 24-well plates, and siRNA-mediated *Hmgcr* KD was performed as described earlier. Cholesterol efflux was carried out as described previously (23).

### Quantification of Oxidative Phosphorylation

The rate of oxygen consumption was measured using a Seahorse Xfe24Extracellular Flux Analyzer (Agilent Technologies) as described previously (22). Briefly, 60 000 GLUTag cells were seeded per well on poly-l-lysine–coated Seahorse XF cell culture plates and oxygen consumption rates were determined as previously described (22).

### Acute and Chronic in Vivo Analyses

Mice aged 12 weeks C57Bl/6j from Janvier Laboratories (RRID: IMSR_RJ:C57BL-6JRJ) were housed in a climate-controlled room (23 °C ± 1 °C) with a 12:12-hour light-dark cycle. Prior to OGTT, mice were fasted for 4 hours and anesthetized using an intraperitoneal (i.p.) injection of Hypnorm/Dormicum (10 μg/L body weight; fentanyl 0.315 mg/mL, fluanisone 10 mg/mL, and midazolam 5 mg/mL) prior to basal blood sampling. A dose of 0.2-mg rosuvastatin was administered orally 1 hour pre OGTT. Basal blood samples were collected by retro-orbital puncture (40 μL), after which 3 g/kg glucose was administered orally (n = 10 per group). Samples were collected in EDTA-coated tubes coated with 0.1 mmol/L Ile-Pro-Ile. Blood samples were collected by retro-orbital puncture at 5, 10, 15, 30, 60, and 90 minutes post challenge and spun at 5000 rpm at 4 °C for 2 minutes. Collected plasma was frozen at −80 °C until analyses for blood glucose and GLP-1. Glucose was measured using an AccuChekAviva glucose meter (Roche), and GLP-1 was measured using the Multi Species GLP-1 Total ELISA (MilliporeSigma). Mice recovered overnight in cages on a heated mat underneath heat lamps.

For chronic treatment, mice were randomly grouped based on weight and injected with saline via i.p. injection for 3 days prior to study commencement. Mice were i.p. injected with either saline or 0.2-mg rosuvastatin in saline for 27 consecutive days. Mice were lightly sedated in a bell jar with Isoflurane (IsoFlo vet 100%, Abbott) and 40 μL of plasma was collected by retro-orbital puncture on days 1, 8, 15, and 22 of the study. On day 27 an OGTT was performed as described earlier. Mice were euthanized on day 28 and the pancreas, liver, and large intestine were flash-frozen in liquid nitrogen or placed in 4% paraformaldehyde in 0.1 M phosphate-buffered saline (pH 7.2). The small intestine was separated into the duodenum, jejunum, and ileum, and flash-frozen in liquid nitrogen or rolled as per the “Swiss roll technique” (24) and placed in 4% paraformaldehyde. After fixation, specimens were dehydrated in alcohol before embedding in paraffin. Paraffin-embedded samples were cut at 6-μm sections and heat-fixed onto coated slides.

### Ethics Statement

The studies in animals and the studies in humans were approved by the regional animal ethics committee in Malmö/Lund and the research ethical review board at Lund University, respectively. A written informed consent was obtained from all study participants.

### Statistical Analyses

All preclinical statistical analyses were performed using GraphPad Prism 10.2.0 (GraphPad Software). Grouped analyses were performed using one-way analysis of variance with a Dunnett multiple comparisons test with a single pooled variance; 2-tailed *t* tests were performed for paired analyses. Data are presented as mean ± SEM.

For clinical samples, Ln-transformations were used to normalize the distribution of nonnormally distributed variables prior to analysis (pre- and post-challenge GLP-1, GIP, insulin, FPG, glucagon, and HOMA-IR). Between-group comparisons were carried out using *t* tests (normally distributed variables) and Mann-Whitney *U* tests (skewed variables) for continuous variables, and χ^2^ tests for binary variables. Linear regression analyses were carried out for associations between statin usage (independent variable) and each of fasting and postchallenge-dependent variables GLP-1, GIP, insulin, glucose, glucagon, and HOMA-IR in unadjusted model 1 and age-, sex-, and body mass index (BMI)-adjusted model 2. The same set of analyses was carried out again with the population split on participants with and without diabetes. Further, correlations between glucose, glycated hemoglobin A_1c_ (HbA_1c_), insulin, and glucagon and incretin measures were assessed using Spearman rank correlation coefficients (ρ) with corresponding *P* values. For a subset of participants with available baseline fasting glucose in the initial MDCS study (n = 1447), we performed a sensitivity analysis using linear regression models adjusting for baseline glucose in addition to other covariates. Baseline gut hormone levels were not available in the MDCS.

All clinical analyses were performed in SPSS Windows 26.0 (SPSS Inc). A 2-tailed *P* value less than .05 was considered statistically significant.

## Results

### Associations Between Statin Use and Fasting Levels of Glucagon-like Peptide 1, Glucose-dependent Insulinotropic Peptide, Insulin, Glucose, Glucagon, and Homeostatic Model Assessment of Insulin Resistance in a Human Population Study

We used the MDCS-CC cohort (12) to test whether statin usage affects GLP-1 plasma levels in humans. Characteristics of the study population are presented in Table 1. Fasting GLP-1 concentrations were higher in statin users compared with nonusers in the whole cohort (*P* < .001), in participants with T2D (*P* < .001), and in participants without T2D (*P* = .023). As anticipated, individuals with T2D exhibited distinctions from those without diabetes in various aspects, such as sex distribution, BMI, utilization of statin medication, as well as levels of incretins, glucose, insulin, and glucagon, except for age (see Table 1). Linear regression analyses adjusted for age, sex, and BMI in the whole cohort showed statistically significant associations between statin usage and increased fasting GLP-1, GIP, insulin, glucose, glucagon, and HOMA-IR (Table 2). We next analyzed participants with T2D and participants without T2D separately. In participants without T2D, statin usage was associated with higher fasting GIP, insulin, glucose, glucagon, and HOMA-IR, but not GLP-1. However, in participants with T2D, statin usage was associated with higher fasting GLP-1 levels, but not with fasting GIP, insulin, glucose, glucagon, and HOMA-IR (see Table 2).

**Table 1.** Characteristics of the study population from the Malmö Diet and Cancer study cardiovascular cohort

	Whole population	Participants without diabetes	Participants with diabetes	*P*
Age, y	72.5 (±5.6)	72.4 (±5.6)	72.9 (±5.2)	.102
Sex, women, n (%)	2183 (29.1)	2014 (60.5)	190 (48.5)	<.001
BMI	26.9 (±4.4)	26.6 (±4.3)	29.3 (±5.2)	<.001
Diabetes status, n (%)	392 (10.6)	—	—	—
Statin treatment, n (%)	1092 (29.6)	855 (25.7)	239 (61.0)	<.001
GLP-1^0 min^, pmol/L	8 (6-10)	8 (6-10)	9 (7-12)	<.001
GLP-1^120 min,^*^a^*, pmol/L	16 (12-21)	16 (12-21)	—	—
GIP^0 min^, pmol/L	41 (30-60)	40 (30-54)	53 (38-78)	<.001
GIP^120 min,^*^a^*, pmol/L	223 (163-294)	223 (163-294)	—	—
Glucose^0 min^, mmol/L	5.9 (5.4-6.5)	5.8 (5.4-6.3)	8.1 (6.9-9.7)	<.001
Glucose^120 min,^*^a^*, mmol/L	6.8 (5.5-8.3)	6.8 (5.5-8.3)	—	—
Insulin^0 min^, mIU/L	8 (5-11)	8 (5-11)	10.4 (6.5-14.4)	<.001
Insulin^30 min,^*^a^*, mIU/L	42 (29-61)	42 (29-61)	—	—
Insulin^120 min,^*^a^*, mIU/L	40 (26-64)	40 (24-64)	—	—
Glucagon^0 min^, pg/mL	77 (64-92)	75 (63-90)	90 (76-108)	<.001
Glucagon^120 min,^*^a^*, pg/mL	80 (58-83)	70 (58-83)	—	—
HOMA-IR	2.0 (1.4-3.1)	1.9 (1.3-2.8)	3.6 (2.3-5.6)	<.001

Values are mean (±SD), median (25th-75th interquartile range), or number (%).

Abbreviations: BMI, body mass index; GIP, glucose-dependent insulinotropic peptide; GLP-1, glucagon-like peptide 1; HOMA-IR, homeostatic model assessment of insulin resistance.

^*a*^Oral glucose tolerance testing was carried out only in participants without diabetes.

**Table 2.** Associations between statin usage and fasting levels of glucagon-like peptide 1, glucose-dependent insulinotropic peptide, insulin, glucose, glucagon, and homeostatic model assessment of insulin resistance

Whole population			Individuals without diabetes		Individuals with diabetes	
**GLP-1**						
	n = 3618		n = 3235		n = 383	
	β	*P*	β	*P*	β	*P*
Model 1	.074	**5.20 × 10^−5^**	.028	.154	**.175**	**.001**
Model 2	.073	**8.90 × 10^−5^**	.029	.144	**.183**	**.001**
**GIP**						
	n = 3476		n = 3091		n = 385	
Model 1	.099	**4.7 × 10^−8^**	.064	**.001**	−.018	.743
Model 2	.079	**1.2 × 10^−5^**	.053	**.007**	−.035	.522
**Insulin**						
	n = 3481		n = 3099		n = 382	
Model 1	.210	**1.5** × **10^−26^**	.207	**2.3 × 10^−23^**	.051	.472
Model 2	.142	**4.2 × 10^−16^**	.165	**8.8 × 10^−20^**	.005	.946
**Glucose**						
	n = 3690		n = 3229		n = 391	
Model 1	.096	**3.8 × 10^−48^**	.047	**3.3 × 10^−21^**	.005	.857
Model 2	.081	**4.3 × 10^−37^**	.040	**8.4** × **10^−17^**	−.001	.976
**Glucagon**						
	n = 3667		n = 3227		n = 390	
Model 1	.097	**1.8 × 10^−22^**	.075	**3.7** × **10^−12^**	.050	.064
Model 2	.072	**4.4 × 10^−14^**	.058	**2.3 × 10^−8^**	.035	.169
**HOMA-IR**						
	n = 3481		n = 3099		n = 382	
Model 1	.307	**5.0 × 10^−43^**	.252	**7.6 × 10^−28^**	.069	.362
Model 2	.225	**8.4 × 10^−31^**	.203	**3.1 × 10^−24^**	.016	.434

Values are unstandardized β coefficients. Values in bold indicate statistically significant associations to the individuals included in the cohort. Model 1: unadjusted. Model 2: adjusted for age, sex, and body mass index.

Abbreviations: GIP, glucose-dependent insulinotropic peptide; GLP-1, glucagon-like peptide 1; HOMA-IR, homeostatic model assessment of insulin resistance.

### Associations Between Statin Usage and Postchallenge Levels of Glucagon-like Peptide 1, Glucose-dependent Insulinotropic Peptide, Insulin, Glucose, and Glucagon

Linear regression analyses adjusted for age, sex, and BMI in participants without T2D showed statistically significant associations between statin usage and higher postchallenge insulin (at both 30 and 120 minutes), glucose, and glucagon, but not with postchallenge GLP-1 and GIP. There were no data available for postchallenge levels in patients with T2D (Table 3).

**Table 3.** Associations between statin usage and postchallenge levels of glucagon-like peptide 1, glucose-dependent insulinotropic peptide, insulin, glucose, and glucagon

Individuals without diabetes		
**GLP-1**		
n = 3189		
	β	*P*
Model 1	−.022	.233
Model 2	−.008	.630
**GIP**		
n = 3056		
Model 1	.027	.149
Model 2	.032	.080
**Insulin at 30 min post challenge**		
n = 2919		
Model 1	.153	**5.8 ×10^−11^**
Model 2	.133	**3.4 × 10^−9^**
**Insulin at 120 min post challenge**		
n = 3056		
Model 1	.322	**6.2 × 10^−28^**
Model 2	.274	**9.2 × 10^−24^**
**Glucose**		
n = 3265		
Model 1	.098	**1.4 × 10^−14^**
Model 2	.081	**9.3 × 10^−11^**
**Glucagon**		
n = 3236		
Model 1	.036	**.001**
Model 2	.028	**.010**

Values are unstandardized β coefficients. Values in bold indicate statistically significant associations. Analyses on postchallenge values were carried out in individuals without diabetes. Model 1: unadjusted. Model 2: adjusted for age, sex, and body mass index.

Abbreviations: GIP, glucose-dependent insulinotropic peptide; GLP-1, glucagon-like peptide 1.

In a sensitivity analysis restricted to 1447 individuals with both baseline and follow-up data, statin use was not significantly associated with subsequent fasting glucose after adjustment for baseline fasting glucose.

In individuals without diabetes, fasting and postchallenge glucose correlated moderately with HbA_1c_, and only weakly with fasting and postchallenge GIP (Supplementary Fig. S1) (25).

### Effect of Statins on GLUTag Cells

**Figure 1.**
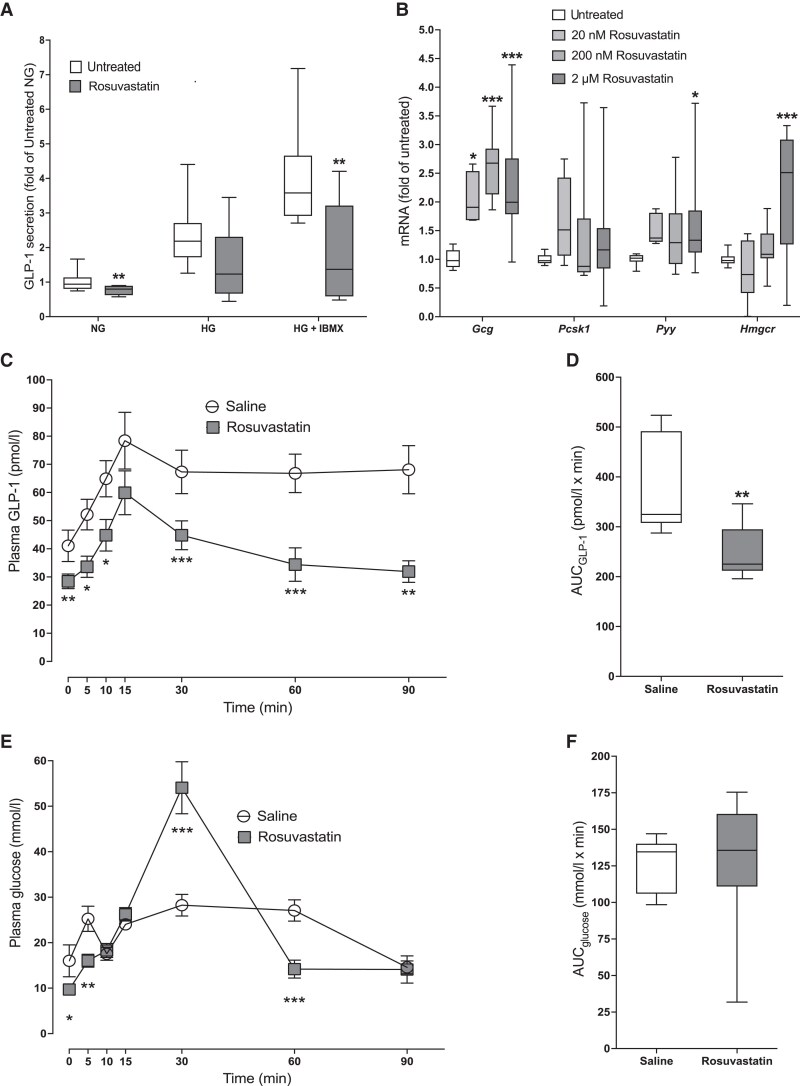
A, A 2-μmol/L dose of rosuvastatin reduces glucagon-like peptide 1 (GLP-1) secretion in response to KRB with no glucose (NG) and 16.7 mmol/L and 10 μmol/L IBMX (HG + IBMX). B, A 2-μmol/L dose of rosuvastatin increases *Gcg*, *Pyy*, and *Hmgcr* expression in GLUTag cells. C to F, A 0.2-mg dose of rosuvastatin administered 1 hour prior to an oral glucose tolerance test (OGTT) in C57Bl6/j mice C, reduces GLP-1 secretion; area under the curve (AUC) is presented in D. E, Plasma glucose levels during the OGTT; AUC is presented in F. OGTTs were performed with 10 mice per group. NG = 0 mM glucose. HG = 16.7 mM glucose. All analyses were performed in duplicate in 6 different passages of GLUTag cells. Data are presented as box and whisker plots showing the minimum and maximum values (bottom and top error bars), the first (bottom of the box) and third (top of the box) quartiles and the median (middle of the box), or as a line graph showing the mean ± SEM. *P less than .05; **P less than .01; and ***P less than .001 compared with either saline or glucose during the same OGTT.

As the analysis in MDCS showed an association between statin use and GLP-1 levels, we next performed functional experiments to test whether statins directly affect GLP-1 secretion and production. First, we used GLUTag cells as a model system and the commonly used statin rosuvastatin as the test substance. RNA-seq confirmed expression of the target for rosuvastatin, *Hmgcr* in these cells (RKPM 4247.5, unpublished data). The addition of 2-µmol/L rosuvastatin caused reduced GLP-1 secretion both in basal (0 mmol/L glucose; 24%; *P* < .01) and stimulatory (16.7 mmol/L glucose + 10 µmol/L IBMX; 55%; *P* < .01) conditions (Fig. 1A). To gain insight into the mechanism behind these effects, we performed RNA-seq of GLUTag cells treated with or without 2-μmol/L rosuvastatin for 24 hours. DESeq2 analysis failed to detect any false discovery rate–corrected differentially expressed genes, but 376 genes were nominally differentially expressed (Supplementary Table S1) (25). GO-term analysis using these genes showed that genes related to cholesterol biosynthetic processes, in particular the mevalonate pathway of cholesterol biosynthesis, were overrepresented among the upregulated genes in rosuvastatin-treated cells (Supplementary Table S2) (25). These genes included but were not limited to *IDI1*, *FDPS*, *MVK*, *HMGCS1*, *MSMO1*, *HMGCR*, *LSS*, *TM7SF2*, *NSDHL*, *MVD*, *DHCR7*, and *FDFT1*. For the downregulated genes, there were no significant GO-terms or clear pattern of genes affected. We confirmed that rosuvastatin treatment upregulated *Hmgcr* messenger RNA (mRNA) expression using qPCR. This was paralleled by increased expression of *Gcg* and *Pyy*, a hormone also expressed in intestinal L cells (26), while no effects were observed on expression of *Pcsk1,* which encodes the proglucagon processing PC1/3 protein (Fig. 1B).

### Acute Effect of Rosuvastatin in Mice

We next assessed the effect of rosuvastatin on GLP-1 secretion during an OGTT in vivo in mice. Lean female C57Bl/6j mice were orally gavaged with 0.2-mg rosuvastatin (27) 1 hour prior to OGTT. This resulted in reduced basal and glucose-stimulated GLP-1 levels (Fig. 1C). Overall, there was a reduction of 35% in the area under the curve (AUC) for GLP-1 (Fig. 1D). Rosuvastatin treatment caused a reduction in basal and 5-minute glucose levels, but a notable 1.9-fold increase in 30-minute glucose, followed by a steep decline in glucose levels at 60 minutes, when 48% lower levels were observed vs controls (Fig. 1E and 1F).

### Long-Term Effect of Rosuvastatin In Vivo

**Figure 2.**
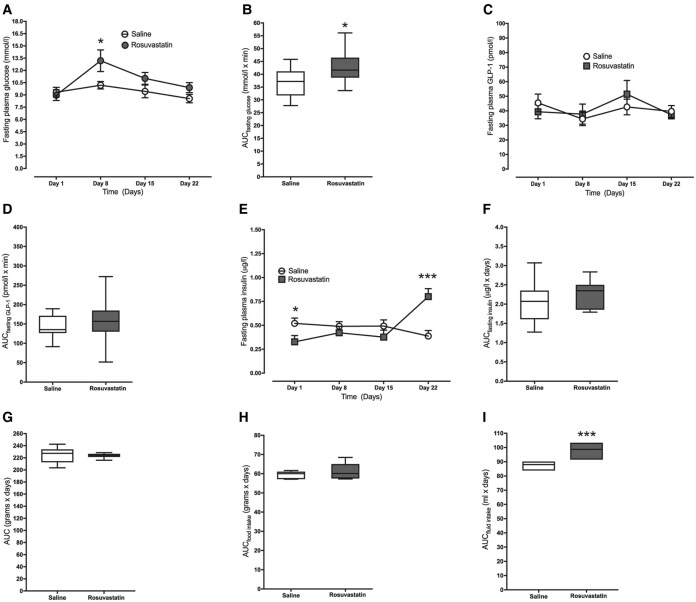
A, Rosuvastatin (0.2 mg) treatment once daily for 27 days causes increased fasting glucose levels; area under the curve (AUC) is presented in B. There were no effects on C, fasting glucagon-like peptide 1 (GLP-1); AUC is presented in D. E, Fasting insulin was increased at day 22, F, but there was no overall change in AUC. Rosuvastatin treatment had no effect on G, body weight or H, food intake but caused I, increased water intake. Data are presented as box and whisker plots showing the minimum and maximum values (bottom and top error bars), the first (bottom of the box) and third (top of the box) quartiles and the median (middle of the box), or as a line graph showing the mean ± SEM. *P less than .05 and ***P less than .001 compared with vehicle-treated mice. Both experimental groups had 10 mice per group.

Having established that rosuvastatin affects GLP-1 secretion in an acute setting, we next addressed the effects of chronic rosuvastatin treatment. We administered 0.2-mg rosuvastatin once daily for 27 days via i.p. injections to lean female C57Bl/6j nondiabetic mice. Rosuvastatin treatment caused elevated FPG levels. In fact, 8 days into the study rosuvastatin-treated mice had 23% higher fasting glucose (Fig. 2A) and there was a 1.2-fold increase in FPG over the course of the study based off AUC (Fig. 2B). Chronic treatment with rosuvastatin had no effect on fasting GLP-1 levels at any time point assessed (Fig. 2C and 2D). Rosuvastatin also affected fasting plasma insulin. Initially the rosuvastatin group had 37% lower fasting plasma insulin, but at day 22 the rosuvastatin group had a 2.1-fold higher fasting plasma insulin (Fig. 2E and 2F). Administration of rosuvastatin had no gross effects on body weight or food intake (Fig. 2G and 2H), but water intake increased (Fig. 2I).

**Figure 3.**
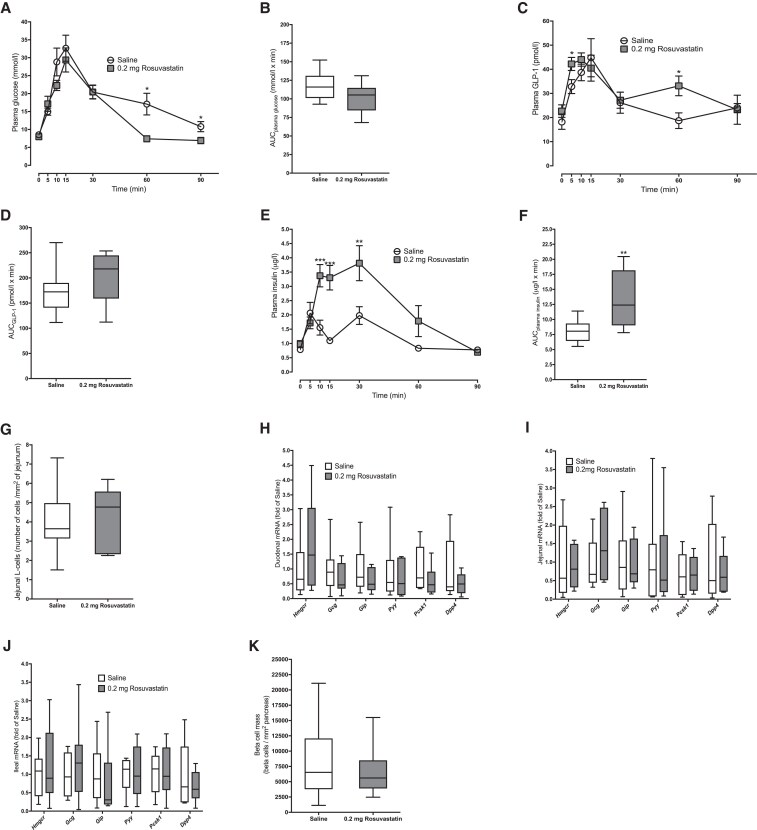
A to F, Oral glucose tolerance test (OGTT) performed after rosuvastatin (0.2 mg) treatment once daily for 27 days. A, Rosuvastatin-treated mice exhibit lower 60- and 90-minute plasma glucose levels, but B, area under the curve (AUC) was unaffected for glucose. C, Rosuvastatin-treated mice exhibit higher 5- and 60-minute plasma glucagon-like peptide 1 (GLP-1) levels, but D, AUC was unaffected for GLP-1. E, Rosuvastatin-treated mice had higher insulin secretion; AUC is presented in F. G, Long-term administration of rosuvastatin had no effect on the number of L cells in the jejunum. H to J, Long-term administration of rosuvastatin had no effect on intestinal messenger RNA expression of *Gcg*, *Gip*, *Pyy*, *Pcsk1*, or *Dpp4* in the H, duodenum; I, jejunum; or J, ileum. K, Long-term administration of rosuvastatin had no effect on β-cell mass. All parameters were assessed in 10 mice per group. Data are presented as box and whisker plots showing the minimum and maximum values (bottom and top error bars), the first (bottom of the box) and third (top of the box) quartiles and the median (middle of the box), or as a line graph showing the mean ± SEM. *P less than .05; **P less than .01; and ***P less than .001 compared with vehicle-treated mice.

To test how long-term administration of 0.2-mg rosuvastatin affects postprandial responses in GLP-1, an OGTT was performed on day 27 of the study. Rosuvastatin-treated mice had lower plasma glucose at 60 and 90 minutes (Fig. 3A), but unaffected AUC for glucose (Fig. 3B). Rosuvastatin-treated mice had increased GLP-1 levels, at 5 and 60 minutes post challenge (Fig. 3C), but unaffected AUC for GLP-1 (Fig. 3D). Rosuvastatin-treated mice also had a marked increase in insulin secretion (Fig. 3E and 3F). Chronic administration of 0.2-mg rosuvastatin had no effect on jejunal L-cell density (Fig. 3G) or expression of *Gcg*, *Gip*, *Pyy*, *Pcsk1*, or *Dpp4* mRNA in the small intestine (Fig. 3H-3J), or on β-cell mass (Fig. 3K).

### Effects of Small Interfering RNA-mediated *Hmgcr* Knockdown in GLUTag Cells

**Figure 4.**
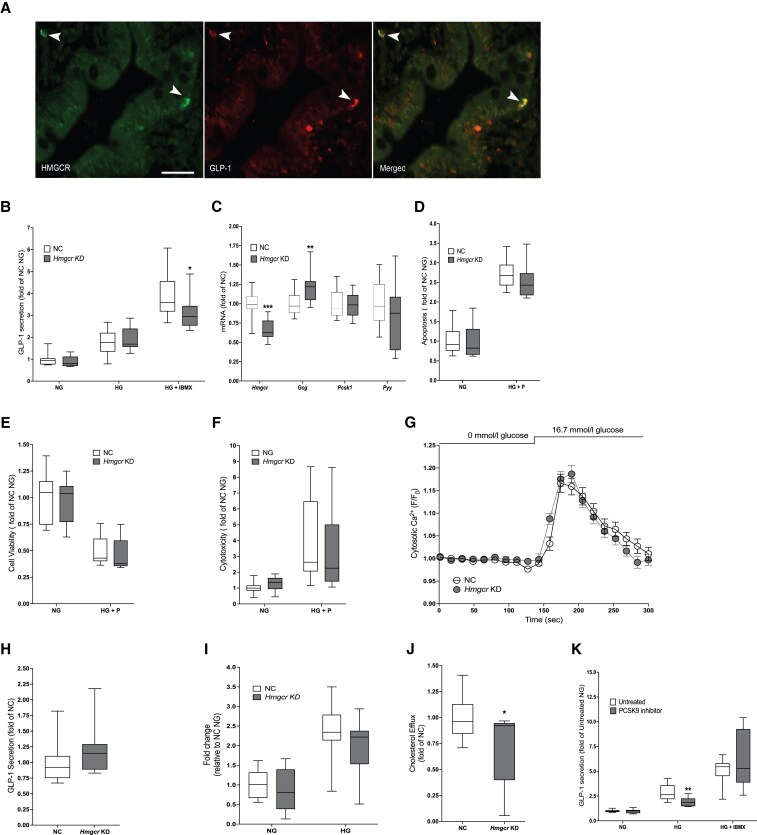
A, Double-immunostaining for HMGCR (green) and glucagon-like peptide 1 (GLP-1) (red) in human jejunal specimen. Arrowheads indicate colocalization. Scale bar = 50μm. B to K, Experiments in GLUTag cells. B, *Hmgcr* knockdown (KD) reduces GLP-1 secretion in response to 16.7 mmol/L glucose with 10 μmol/L IBMX. C, *Hmgcr* KD increases *Gcg* gene expression but has no effect on messenger RNA expression of *Pcsk1* or *Pyy*. D to F, The effects of *Hmgcr* KD on D, apoptosis; E, cell viability; and F, cytotoxicity in response to Dulbecco’s modified Eagle’s medium (DMEM) with no glucose (NG) or 25-mmol/L glucose and 500-μmol/L palmitate (HG + *P*) in GLUTag cells. G, *Hmgcr* KD has no effect on intracellular calcium concentrations. H, *Hmgcr* KD has no effect on GLP-1 secretion in response to 50-mmol/L KCl. I, *Hmgcr* KD has no effect on glucose uptake in response to NG or HG in GLUTag cells. J, *Hmgcr* KD reduces cholesterol efflux. K, A dose of 1-μmol/L of the PCSK9 inhibitor PF-06446846 hydrochloride reduces GLP-1 secretion from GLUTag cells in response to 16.7 mmol/L glucose (HG). Data are presented as box and whisker plots showing the minimum and maximum values (bottom and top error bars), the first (bottom of the box) and third (top of the box) quartiles and the median (middle of the box), or as a line graph showing the mean ± SEM. NG = 0-mM glucose. HG = 16.7-mM glucose. All analyses were performed in duplicate in 6 different passages of GLUTag cells. *P less than .05; **P less than .01; and ***P less than .001 compared to negative control (NC) or untreated cells.

As we found rosuvastatin to affect GLP-1 production and secretion, we next tested whether we could replicate this by targeting *Hmgcr* with siRNA. First we confirmed HMGCR expression in human intestinal L cells using immunohistochemistry (Fig. 4A). siRNA-mediated *Hmgcr* KD had no effect on GLP-1 secretion at 0 mmol/L or 16.7 mmol/L glucose. However, similarly to rosuvastatin treatment, *Hmgcr* KD reduced GLP-1 secretion stimulated with a combination of 16.7 mmol/L glucose and 10 µmol/L IBMX (28) (Fig. 4B; *P* < .05). *Hmgcr* KD caused a 1.2-fold increase in *Gcg* gene expression (*P* < .001), while *Pcsk1* and *Pyy* expression were unaffected (Fig. 4C). Therefore, it is unlikely that the observed reduction in GLP-1 secretion was due to effects on *Gcg* expression. We performed experiments aiming to provide mechanistic insight into the observed effect of *Hmgcr* KD on GLP-1 secretion. First, we used an ApotoxGlo Triplex assay and found that *Hmgcr* KD had no effect on apoptosis (Fig. 4D), cell viability (Fig. 4E), or cytotoxicity (Fig. 4F). Next, we assessed whether *Hmgcr* KD affected intracellular calcium concentrations, which would affect exocytosis from GLUTag cells. *Hmgcr* KD had no effect on glucose-induced intracellular calcium concentrations (Fig. 4G). This was further corroborated by a lack of effect of *Hmgcr* KD on GLP-1 secretion in response to 50-mmol/L KCl (Fig. 4H). Furthermore, *Hmgcr* KD had no effect on glucose uptake (Fig. 4I), but reduced cholesterol efflux capacity by 34% (Fig. 4J; *P* < .05). To test whether the effect of HMGCR on GLP-1 secretion involves the mitochondria, we employed a Seahorse XF Cell Mito Stress test (Supplementary Fig. S2) (25). While *Hmgcr* KD cells respired similarly to control cells in response to raised glucose, maximal respiration rates were increased 1.3-fold (Supplementary Fig. S2C; *P* < .05) (25) after *Hmgcr* KD. All other respiratory parameters measured demonstrated no statistically significant differences (see Supplementary Fig. S2) (25). Thus, the effect of *Hmgcr* KD on GLP-1 secretion is unlikely explained by actions on stimulus secretion coupling, cell proliferation, or cell death.

### Pcsk9 Inhibition Reduces Glucagon-like Peptide 1 Secretion in GLUTag Cells

We next tested the possibility that the effect of rosuvastatin on GLP-1 secretion is secondary to lowering of intracellular low-density lipoprotein (LDL). To this end, we employed a commercially available PCSK9 inhibitor, PF-06446846 hydrochloride, which inhibits LDL independently of HMGCR (29). Treatment with PF-06446846 hydrochloride reduced GLP-1 secretion by 32.3% compared with untreated cells in response to 16.7 mmol/L glucose (Fig. 4K).

## Discussion

Despite great interest in GLP-1 and the successful use of incretin-based medication as treatments for T2D, it is not well known how GLP-1 secretion is regulated in humans. Here we show that rosuvastatin and its target molecule HMGCR regulate GLP-1 secretion and production. In the short-term, inhibition of HMGCR expression with siRNA or chemical inhibition with rosuvastatin has an acute inhibitory effect on GLP-1 secretion in an L-cell model, as well as during an OGTT in mice. On the other hand, long-term daily rosuvastatin treatment caused an increased postprandial GLP-1 response in normoglycemic mice. We also assessed the effect of statin use on glucose and glucoregulatory hormones in a large human cohort (30). Indeed, statin use was associated with increased fasting, but not postprandial, GLP-1 levels. When the analysis was stratified for disease state, no such association was observed in individuals without T2D, but in participants with T2D, statin use was associated with higher fasting GLP-1 levels. Overall, correlations of GLP-1 with glucose and HbA_1c_ were weak both in diabetic and nondiabetic participants. These results suggest that our human data do not support a direct link between GLP-1 concentrations and glycemia. However, they should be interpreted with caution, as fasting GLP-1 levels may not adequately reflect the hormone's postprandial role in glucose regulation. We also replicated previously reported findings (31) of worsened glycemic control in statin users. Thus, statin use was associated with higher glucose levels in the overall population and individuals without T2D, with no statistically significant association observed in participants with T2D. This observation gains support from our mouse data showing elevated postchallenge glucose levels after rosuvastatin treatment. Interestingly, the relationship between statin use and GIP levels did not mirror the relationship observed between statin use and GLP-1 levels. In the overall population, as well as in normoglycemic subjects, statin use was associated with higher GIP levels. However, in subjects with T2D, no association with statin use and GIP levels was evident. These apparently divergent results suggest that GLP-1 responses to statins may depend on the duration of treatment and underlying metabolic state. We therefore interpret GLP-1 changes as potentially contributing to short-term fluctuations in glucose metabolism, while the long-term diabetogenic risk of statins is likely mediated by additional pathways beyond GLP-1.

We made efforts to decipher the cellular mechanisms behind the inhibitory effect of rosuvastatin on GLP-1 secretion. As we found rosuvastatin treatment and *Hmgcr* KD to increase *Gcg* mRNA, the effect is unlikely a consequence of altered production of GLP-1. Furthermore, we found no effect of *Hmgcr* KD on glucose uptake, intracellular Ca^2+^ levels, cell viability, or cell death. *Hmgcr* KD caused a moderate increase in maximal respiration. This would rather be associated with an increase in GLP-1 secretion, and therefore we believe that the inhibitory effect of HMGCR on GLP-1 secretion does not involve the mitochondria. As expected, *Hmgcr* KD caused reduced cholesterol efflux. On the same note, and favoring a direct role of cholesterol per se in regulation of GLP-1 secretion, a PCSK9 inhibitor, which lowers cholesterol via a different mechanism (29), also reduced GLP-1 secretion. Based on this observation and the lack of effect on the aforementioned readouts, we propose that the inhibitory effect of HMCGR on GLP-1 secretion is explained by the LDL cholesterol lowering. This notion also gains support from our findings that the effects of rosuvastatin on the GLUTag cell transcriptome were limited to cholesterol-related genes. Cholesterol is a crucial component of the cell membrane and reduced intracellular content has been shown to hamper insulin secretion from β cells, likely as a consequence of perturbed formation of fusion pores (32). Such alterations may not be captured by altered mRNA expression, and further studies are needed to pinpoint the exact mechanisms behind the observed effects of statin treatment and *Hmgcr* KD on GLP-1 secretion.

Our in vivo studies are limited in that they were performed only in female mice and that the chronic study was performed in normoglycemic mice without the confounding influences of obesity or T2D. Chronic rosuvastatin treatment (i.p.) in mice caused increased postprandial GLP-1 secretion. This is contrary to the effect in the acute experiments, in which rosuvastatin was given orally. There is no ready explanation for this, and the potential influence of administration routes or duration of administration needs further investigation. These different administration routes were used as we did not have ethical permission to orally administer rosuvastatin chronically but did have permission to orally administer rosuvastatin acutely.

Furthermore, our human studies are limited in that statin use was not stratified for different types of prescribed statins and, for ethical reasons, lack of OGTT data for individuals with T2D. In sensitivity analyses, baseline fasting glucose was available in only a subset of participants (n = 1447), and statin use was not associated with subsequent fasting glucose after adjustment for baseline values. While this suggests that baseline glycemia explains part of the observed associations, the limited sample size means that these findings should be interpreted with caution and do not negate the overall results of the study. In the MDCS-CC cohort, we observed higher fasting GLP-1 concentrations among participants with T2D compared with those without diabetes. Although the incretin effect is typically reduced in T2D (33), prior studies have reported heterogeneous results, with some describing preserved or even elevated fasting GLP-1 levels (34). Such elevations may reflect compensatory increases in secretion or altered degradation of GLP-1 in the setting of chronic metabolic stress and concomitant therapies. Importantly, the absence of OGTT data for the diabetes subgroup limits mechanistic interpretation, and we acknowledge that this restricts direct conclusions about the role of GLP-1 in mediating statin-induced dysglycemia. In contrast, among nondiabetic individuals undergoing OGTT, statin use was not associated with differences in GLP-1, suggesting that changes in GLP-1 alone are unlikely to account for the diabetogenic effects of statins. Also, correlation analyses indicate that fasting GLP-1 concentrations were not significantly associated with glycemia, and that postchallenge GLP-1 correlated only weakly and inversely with glucose, insulin, and insulin resistance indices. Thus, our human data do not support a direct link between GLP-1 concentrations and glucose homeostasis, reinforcing the notion that statin-induced diabetogenicity is multifactorial and not solely mediated by GLP-1.

In summary, we have shown that statin use is associated with GLP-1 levels in humans. In experimental models acute rosuvastatin treatment or *Hmgcr* KD lowered GLP-1 secretion, likely via its action on intracellular cholesterol levels. Our experimental data may partly explain the clinically observed mild hyperglycemia associated with statin use. We have also shed light on altered responses to rosuvastatin in patients with T2D. Given the negative acute effect on GLP-1 secretion, we recommend the regular monitoring and control of blood glucose levels following prescription of rosuvastatin.

## Data Availability

All data are available from the corresponding author on request.

## Abbreviations

AUC: area under the curve
BMI: body mass index
ELISA: enzyme-linked immunosorbent assay
FPG: fasting plasma glucose
GIP: glucose-dependent insulinotropic peptide
GLP-1: glucagon-like peptide 1
GO: Gene ontology
HbA_1c_: glycated hemoglobin A_1c_
HMGCR: 3-hydroxy-3-methyl-glutaryl-coenzyme A reductase
HOMA-IR: homeostatic model assessment of insulin resistance
i.p.: intraperitoneal
KD: knockdown
LDL: low-density lipoprotein
MDCS: Malmö Diet and Cancer study
MDCS-CC: Malmö Diet and Cancer study cardiovascular cohort
mRNA: messenger RNA
OGTT: oral glucose tolerance test
qPCR: quantitative real-time polymerase chain reaction
RNA-seq: RNA sequencing
siRNA: small interfering RNA
T2D: type 2 diabetes
